# Vitamin D in Reproductive Health Disorders: A Narrative Review Focusing on Infertility, Endometriosis, and Polycystic Ovarian Syndrome

**DOI:** 10.3390/ijms26052256

**Published:** 2025-03-03

**Authors:** Ximena A. van Tienhoven, Jimena Ruiz de Chávez Gascón, Gabriela Cano-Herrera, José Antonio Sarkis Nehme, Ariela A. Souroujon Torun, Maria Fernanda Bautista Gonzalez, Felipe Esparza Salazar, Ana Sierra Brozon, Eder Gabriel Rivera Rosas, Dante Carbajal Ocampo, Ramiro Cabrera Carranco

**Affiliations:** 1 Centro de Investigación en Ciencias de la Salud (CICSA), Facultad de Ciencias de la Salud, Universidad Anáhuac México Norte, Lomas Anáhuac, Huixquilucan 52786, Estado de México, Mexicojimerdch@gmail.com (J.R.d.C.G.); jose.sarkis@anahuac.mx (J.A.S.N.); arisourou2000@gmail.com (A.A.S.T.); mariafrenandabg19@gmail.com (M.F.B.G.); felipe.esparzas69@anahuac.mx (F.E.S.); 2 Escuela de Ciencias de la Salud, Universidad Anáhuac Puebla, Orión Norte S/N, La Vista Country Club, San Andrés Cholula 72810, Puebla, Mexico; gabriela.canohe@anahuac.mx; 3 Departamento en Cirugía Pélvica, Doyenne High Quality and Multidisciplinary Treatment Center for Endometriosis, Ciudad de México 06700, Mexico; anag.sierrab@gmail.com (A.S.B.); drriveragyo@gmail.com (E.G.R.R.); consultoriooncogine@gmail.com (D.C.O.)

**Keywords:** vitamin D, reproductive health, fertility, infertility, endometriosis, polycystic ovarian syndrome

## Abstract

Vitamin D (VD) is a fat-soluble steroid hormone with essential physiological functions beyond calcium and bone metabolism. In recent years, its role in women’s reproductive health has gained attention, influencing ovarian function, follicular development, endometrial receptivity, and steroid hormone regulation. VD deficiency has been linked to reproductive disorders such as polycystic ovarian syndrome (PCOS), endometriosis, and infertility. Studies indicate that up to 40–50% of healthy pregnant women have insufficient VD levels, which may contribute to adverse pregnancy outcomes and reduced fertility. With growing evidence connecting VD to reproductive health, this review examines its molecular and endocrine mechanisms in fertility, endometriosis, and PCOS. It explores VD’s therapeutic potential and its implications for improving clinical approaches and future research in reproductive medicine. Maintaining adequate VD levels is crucial for ovarian function, immune modulation in reproductive tissues, and overall fertility. Its deficiency is associated with insulin resistance, hormonal imbalances, and inflammatory processes, which contribute to reproductive pathophysiology. Establishing reference values for VD in reproductive medicine is essential for optimizing fertility treatments and improving clinical outcomes. This review synthesizes current research on VD’s role in reproductive health and highlights the need for further investigation into its therapeutic applications.

## 1. Introduction

VD is a fat-soluble substance essential for overall health, extending beyond its well-known role in bone metabolism to influence various physiological processes. The biologically active form, calcitriol (1,25(OH)2D3), exerts its effects through both genomic and non-genomic mechanisms [1]. Genomically, VD binds to the cytoplasmic vitamin D receptor (VDR), while non-genomic actions occur via interactions with membrane-bound VDR. These receptors are distributed throughout the human body, including reproductive tissues in both males and females [2,3].

In the male reproductive system, VDR is expressed in Sertoli and Leydig cells, germ cells, developing spermatozoa, and mature sperm. In females, VDR is present in the uterus, ovaries, and reproductive tract, as well as in the placenta during pregnancy [4]. Given its widespread distribution, VD plays a pivotal role in reproductive health by regulating processes such as ovarian steroidogenesis, folliculogenesis, spermatogenesis, and the acrosome reaction. Moreover, VD levels have been correlated with sperm quality and ovarian reserve, underscoring its relevance in fertility [5]. Clinical evidence, including randomized controlled trials (RCTs), cohort studies, and meta-analyses, supports the potential therapeutic role of VD in reproduction-related complications. In women with PCOS, RCTs and meta-analyses have shown that VD supplementation improves insulin sensitivity, reduces androgen levels, and enhances ovulatory function [6]. In endometriosis, observational studies and RCTs suggest that VD has anti-inflammatory properties that may alleviate pelvic pain and modulate immune responses [7].

Deficiencies in VD have been implicated in reproductive disorders, including PCOS, endometriosis, and infertility. Observational studies suggest that inadequate VD levels may negatively impact reproductive function by influencing hormonal balance, follicular development, and implantation processes [8]. Additionally, VD plays a role in conception and placental development, emphasizing its significance in sustaining reproductive health [4].

VD plays a multifaceted role in fertility, with studies indicating that adequate levels are associated with improved ovarian reserve and enhanced outcomes in assisted reproductive technologies like in vitro fertilization (IVF) [9]. Conversely, VD deficiency has been linked to reduced anti-Müllerian hormone (AMH) levels, a key marker of ovarian reserve, and decreased fertility potential [10]. In PCOS, a prevalent endocrine disorder marked by hyperandrogenism, ovulatory dysfunction, and polycystic ovarian morphology, 67–85% of affected women exhibit serum 25-hydroxy VD levels below 20 ng/mL, correlating with insulin resistance, menstrual irregularities, hyperandrogenism, and heightened cardiovascular risk factors [11,12]. VD supplementation has demonstrated potential in mitigating these manifestations by improving menstrual regularity, enhancing folliculogenesis, and lowering serum testosterone levels, making it a promising adjunctive therapy in PCOS management. Regarding endometriosis, a chronic condition characterized by the ectopic growth of endometrial-like tissue, VD’s role remains complex. Some studies suggest that VD modulates immune responses and inflammation, potentially influencing disease progression, while others find no significant effect of VD supplementation on symptoms [13,14]. Clinical studies have looked at VD’s impact on pregnancy outcomes in individuals with reproductive-related diseases like endometriosis. Observational studies and RCTs suggest that VD supplementation may improve fertility by reducing inflammatory markers and enhancing endometrial receptivity, potentially increasing implantation and pregnancy rates [8]. Moreover, VD can be used in conjunction with other treatments, such as hormonal therapy and assisted reproductive technologies, to optimize outcomes [15]. However, findings remain inconclusive, and further large-scale trials are needed to establish definitive clinical recommendations.

Given the increasing evidence linking VD to reproductive function and various gynecological disorders, this review aims to critically evaluate the molecular and endocrine mechanisms through which VD influences fertility, endometriosis, and PCOS. By synthesizing recent findings, this analysis seeks to clarify VD’s potential as a therapeutic target for improving reproductive health and fertility outcomes. Understanding these mechanisms is crucial for optimizing clinical approaches and guiding future research on VD supplementation in reproductive medicine.

## 2. Methodology

This narrative review aims to synthesize current knowledge on the role of VD in reproductive health, focusing on its molecular and endocrine mechanisms in infertility, endometriosis, and PCOS.

A comprehensive literature search was conducted using PubMed, Scopus, and Google Scholar, prioritizing studies published in English and Spanish over the last 10 years to ensure the inclusion of updated and relevant research. The search strategy included the keywords “Vitamin D”, “polycystic ovary syndrome”, “PCOS”, “reproductive health”, “fertility”, “infertility”, and “endometriosis”, applying Boolean operators to refine the selection process.

Studies were included if they were published in peer-reviewed journals, written in English or Spanish, had full-text availability, and examined the relationship between VD and reproductive health disorders. Conversely, studies that did not directly assess VD in reproductive health disorders, as well as preprints, unpublished manuscripts, and those with insufficient data or no full-text access, were excluded. Although priority was given to recent publications, older references were included when they contributed essential findings to understanding VD’s role in reproductive health.

## 3. Vitamin D Structure and Metabolism

VD is a fat-soluble steroid hormone with diverse physiological roles. Among its functions, VD acts as an immunomodulator and anti-proliferative mediator while also playing a pivotal role in maintaining calcium, magnesium, and phosphate homeostasis [16]. Recent studies report that up to 40–50% of healthy pregnant women exhibit deficient serum VD levels (≤20 ng/mL), despite concentrations below 30 ng/mL now being recognized as inadequate [17]. VD deficiency is associated with a range of adverse outcomes during pregnancy, including congenital abnormalities, and is a notable risk marker for complications. Furthermore, inadequate VD levels have been linked to fertility challenges and reduced success rates in IVF [16,17].

The primary source of VD synthesis is cutaneous production, accounting for approximately 90% of total VD through ultraviolet (UV) light exposure, while dietary intake contributes the remaining 10% [18]. Foods with high VD concentrations include fatty fish, beef liver, cheese, egg yolks, and fortified milk [19]. Lower amounts of VD are found in mushrooms, cereals, chicken, beef, and various vegetables. The recommended daily intake is 600 IU for individuals under 70 years old and 800 IU for those over 70 [20].

VD is a steroid hormone with two primary forms: vitamin D2 (D2) and vitamin D3 (D3). D3 is synthesized in the skin from 7-dehydrocholesterol (7-DHC) upon UVB exposure, while D2 is derived from ergosterol in plants and fungi, typically obtained through dietary sources [21,22]. Both forms of VD circulate in the bloodstream bound to the vitamin D-binding protein (DBP) and undergo sequential hydroxylation in the liver and kidneys.

In the liver, D2 and D3 are hydroxylated by 25-hydroxylase to form 25-hydroxyvitamin D (25-OH-VD or calcifediol), the major circulating form with a half-life of approximately 14 days [20]. Calcifediol is subsequently hydroxylated by 1-alpha-hydroxylase in the kidneys, producing the biologically active metabolite 1,25-dihydroxyvitamin D (1,25-OH-2D or calcitriol). This active form plays a crucial role in calcium and phosphate homeostasis and regulates cellular processes integral to reproductive health [23,24]. Notably, 1-alpha-hydroxylase is also expressed in extra-renal tissues, including the ovaries, brain, mammary glands, prostate, and colon, allowing localized synthesis of active VD [25,26].

Calcitriol exerts its physiological effects by binding to the VD receptor (VDR) in target tissues, such as the uterus and ovaries, to modulate gene expression and cellular functions. Its actions are essential for reproductive health, influencing calcium absorption, steroidogenesis, and immune modulation. These functions are particularly relevant in fertility and endometriosis, where VD impacts ovarian function, implantation, and inflammation [27].

The degradation of VD metabolites occurs through two primary pathways: the C24 oxidation and C23 lactone pathways. These pathways, mediated by CYP24A1 (24-hydroxylase), convert calcifediol and calcitriol into their inactive forms, such as calcitroic acid, which are excreted in bile and feces. Tight regulation of these metabolic processes ensures a balance of calcitriol levels, preventing toxicity and maintaining calcium homeostasis, which is particularly crucial during pregnancy and in conditions such as endometriosis [23,24].

The hormonal form of VD, 1,25(OH)2D, exhibits paracrine and autocrine functions across a range of tissues, including the skin, immune system, parathyroid gland, intestinal epithelium, hypothalamic–pituitary axis, prostate, and breast. These biological actions are mediated by the VDR, which is widely distributed in cells, including those within the reproductive system [22]. The interaction of the hypothalamic–pituitary axis with VDR underscores a potential regulatory role in reproductive physiology. This axis coordinates reproductive function through hormonal signaling pathways, with the hypothalamus releasing hormones that stimulate the pituitary gland to secrete reproductive hormones targeting the gonads [28].

**Figure 1:**
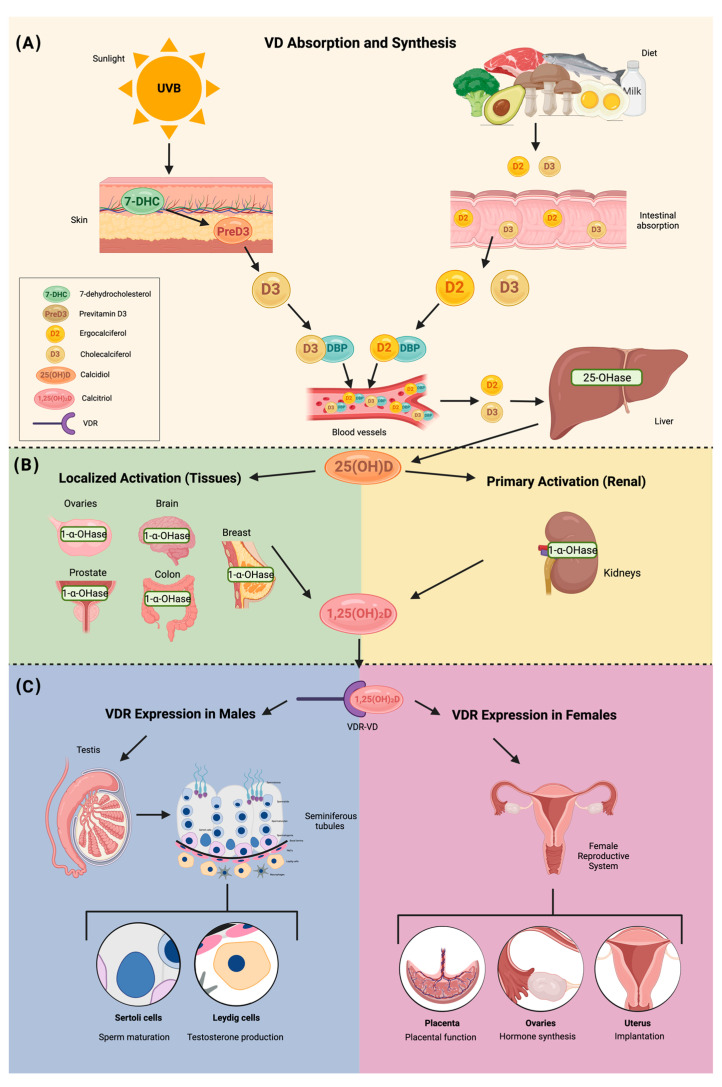
The role of VD in synthesis, activation, and reproductive health. This figure illustrates the role of VD in synthesis, activation, and reproductive health. (**A**) VD absorption and synthesis: VD is obtained from UVB exposure and dietary sources, leading to the formation of cholecalciferol (D3) and ergocalciferol (D2), which undergo hydroxylation in the liver and kidneys to become the active form, calcitriol (1,25(OH)_2_D). (**B**) Activation: Calcitriol is synthesized in the kidneys and locally in various tissues, including the ovaries, prostate, brain, colon, and breast, through 1-α hydroxylase activity. (**C**) VDR expression in reproductive health: In males, VDR is present in Sertoli and Leydig cells, influencing sperm maturation and testosterone production. In females, VDR plays a role in placental function, ovarian hormone synthesis, and uterine implantation, processes that are often disrupted in endometriosis and PCOS, contributing to infertility.

VDR expression is notably prevalent in the female reproductive system, including the ovaries, uterus, placenta, and endometrium. Within ovarian follicles, VDR facilitates the physiological actions of 1,25(OH)2D3, underscoring its critical involvement in gonadal function [22,28]. These findings highlight the direct implications of VD in fertility and broader aspects of reproductive health, establishing its significance in gynecological endocrinology and metabolism (Figure 1).

The assessment of VD status typically involves measuring serum 25-hydroxyvitamin D [25(OH)D], which is widely recognized as the most accurate biomarker for determining VD sufficiency [4]. Despite its widespread use in both clinical and research contexts, the techniques employed to detect this biomarker differ significantly in terms of accuracy, cost, and accessibility.

In clinical environments, automated immunoassays, such as chemiluminescence immunoassays (CLIAs) and enzyme-linked immunosorbent assays (ELISAs), are the most commonly utilized methods. These approaches are favored for their affordability, widespread availability, and ability to deliver quick results, making them ideal for routine healthcare applications [5]. Nevertheless, inconsistencies in assay performance and the possibility of cross-reactivity with other VD metabolites can compromise their reliability, especially when measuring low concentrations of VD [23].

In contrast, research settings often rely on liquid chromatography–tandem mass spectrometry (LC-MS/MS), which is regarded as the gold standard due to its superior precision, accuracy, and capacity to distinguish between D2 and D3 [1]. However, the high cost, need for specialized equipment, and requirement for technical expertise restrict its use primarily to research laboratories, making it less feasible for routine clinical use [8].

To address the inconsistencies between these methods, global initiatives like the VD Standardization Program (VDSP) have been established to standardize measurement techniques across laboratories. These efforts aim to enhance comparability between laboratories and support more reliable clinical decision-making [29].

In summary, while both clinical and research settings rely on 25(OH)D measurements, the methods employed vary, with LC-MS/MS preferred in research for its precision and immunoassays favored in clinical practice for their practicality. Continued standardization initiatives are crucial for improving the consistency and reliability of VD assessments across diverse settings.

## 4. Vitamin D Deficiency

VD deficiency is a global health concern, with prevalence rates varying by geographic region, age, and other socio-demographic factors. VD deficiency is multifactorial; the main cause has been found to be low sun exposure, and decreased UVB absorption occurs when the temperature is below 33 °F, making it challenging for many people to get enough sun exposure below this temperature. Melanin is an undeniable factor partaking in VD deficiency, as it reduces the effectiveness of VD production by absorbing UVB, resulting in darker-skinned individuals needing 5–10 times more sun exposure to reach their VD goals. Sunscreen is another major factor, as application of sunscreen with a sun protector factor (SPF) of 30 or more decreases VD production by 97.5% [30]. Elevated body mass index (BMI) and specific ethnicities, particularly Asian and African American populations, have also been identified as significant risk factors for VD deficiency, with Asian individuals at particularly high risk [21]. In a systematic review and meta-analysis including almost 750,000 subjects, it was evident that Asian populations had a high prevalence of VD deficiency; regarding this phenomenon, the study found factors such as age, altitude, regions, and specific diseases to be some of the main contributors. Nonetheless, it has also been found that Asian populations consume lower amounts of VD-rich foods as they are less available in such areas, and they tend to wear sunscreen which decreases UVB to penetration into the skin [31,32,33].

Furthermore, the literature observes that a deficiency in VD disrupts calcium and parathyroid hormone (PTH) balance, may lead to problems with oocyte maturation, egg development, and fertilization in healthy women, as well as menstrual irregularities, and halt follicular development [34]. The VD system’s role in regulating calcium is also essential for proper sperm motility and the acrosome reaction. Furthermore, VD exerts effects beyond calcium regulation. In males, it decreases triglyceride levels and enhances lipase activity in sperm, meeting the energetic demands needed for sperm capacitation. In females, VD is involved in placental steroid production and the decidualization process of the endometrium, therefore upholding fertility. Moreover, this system helps control key hormones such as human placental lactogen, estradiol, human chorionic gonadotropin, and progesterone, which are crucial for maternal immune tolerance and for managing uteroplacental neovascularization and blood flow [34].

In addition to its direct impact on reproductive function, VD deficiency is frequently associated with other metabolic and autoimmune conditions that may further contribute to infertility. Insulin resistance, a hallmark of metabolic syndrome and PCOS, has been linked to VD deficiency and is known to disrupt ovulatory function and endometrial receptivity. VD plays a role in glucose metabolism and insulin sensitivity, suggesting that its deficiency may exacerbate insulin resistance and, consequently, fertility-related complications [11,12].

Similarly, chronic autoimmune thyroiditis is a well-recognized cause of hypothyroidism, which is a significant contributor to infertility. VD is believed to have immunomodulatory effects that influence thyroid autoimmunity, with studies indicating an inverse relationship between VD levels and thyroid autoantibodies. VD deficiency could worsen thyroid dysfunction and further impair fertility outcomes [35]. Celiac disease, another autoimmune condition associated with infertility, is characterized by chronic intestinal inflammation that leads to malabsorption of essential nutrients, including VD. Women with untreated celiac disease often present with menstrual irregularities, anovulation, and an increased risk of pregnancy complications. Given the essential role of VD in immune regulation and intestinal health, its deficiency in these patients may contribute to both systemic inflammation and reproductive dysfunction [36].

Beyond autoimmune disorders, other micronutrient deficiencies, such as iron deficiency, may further compound the negative effects of VD deficiency on fertility. Iron is crucial for oocyte quality, implantation, and placental function, and its deficiency is commonly observed in individuals with chronic inflammation, malabsorption syndromes, or heavy menstrual bleeding—conditions that frequently coexist with VD deficiency [37]. The combined effect of these nutritional deficiencies may create an unfavorable reproductive environment, emphasizing the need for a broader approach when evaluating infertility in patients with VD deficiency.

A comprehensive epidemiological study conducted across Mexico, Chile, and Brazil revealed that the Mexican population exhibited the highest prevalence of VD deficiency, with 67% of postmenopausal women affected [20]. In particular, individuals with obesity have a lower bioavailability of VD due to its sequestration in adipose tissue, which leads to suboptimal circulating levels [24]. These findings are concerning as VD is essential not only for skeletal health but also for optimal reproductive function [38]. The broad epidemiological data indicate that anyone, regardless of their health status or demographic characteristics, may be vulnerable to VD deficiency, making it an important public health concern globally [23,39].

## 5. Correlation Between Vitamin D and Ovarian Reserve, Steroidogenesis, and Follicular Development

VD exhibits anti-inflammatory and immunomodulatory effects; it has been shown to contribute to immune regulation by suppressing pro-inflammatory cytokines, including tumor necrosis factor-alpha (TNF-α), interleukin-1 (IL-1), and IL-6, while enhancing the expression of anti-inflammatory cytokines [40,41]. A significant correlation between VD levels and follicular development has been observed, suggesting that VD is essential for proper ovarian function and fertility [42].

In particular, research by Safaei et al. (2020) demonstrated that VD affects the expression of crucial genes involved in follicular development and steroidogenesis in human granulosa cells. The study reported that the presence of VD in granulosa cell cultures increased the mRNA expression of AMH and the follicle-stimulating hormone (FSH) receptor gene, both of which play pivotal roles in follicular maturation and selection [43,44]. Moreover, the presence of VDR in ovarian tissues has been confirmed, and stimulating granulosa cells with calcitriol exhibited increased production of important reproductive hormones such as progesterone, estradiol, and estrone [45,46]. These findings collectively suggest that VD has a critical role in regulating follicular development and steroid hormone production.

Additionally, a correlation has been observed between VD concentrations in serum and follicular fluid, indicating that VD may be crucial for follicular maturity and development [42]. A systematic review and meta-analysis conducted by Chu et al. (2018) concluded that women undergoing assisted reproductive treatments who have adequate VD levels have a higher birth rate compared to those who are VD insufficient [47]. This emphasizes the role of adequate VD concentrations in supporting reproductive success during assisted reproduction treatments [47,48].

Recent studies have also explored the influence of VD levels on the expression of transcription factors critical for reproductive health. The HOXA10 and HOXA11 genes, which play key roles in the development of reproductive tissues, are expressed in the endometrial stromal cells of adult women [49,50]. These transcription factors are essential for the success of embryo implantation and fertility, as they facilitate continuous tissue proliferation and differentiation [50]. Research by Shilpasree et al. (2022) revealed that adequate VD levels are associated with higher expression of HOXA10 mRNA, particularly during the luteal phase of the menstrual cycle, which is crucial for implantation and endometrial receptivity [51]. In contrast, women with VD deficiency or insufficiency exhibit reduced expression of HOXA10, potentially impairing reproductive outcomes [52].

Further supporting these findings, Ozkan et al. (2010) conducted a prospective cohort study that showed that women with higher VD levels in their follicular fluid were significantly more likely to achieve successful implantation and pregnancy, with gestational sacs detected on ultrasonography, whether intrauterine or ectopic [53]. Similarly, a retrospective study by Rudick et al. (2014) found that VD insufficiency was linked to lower clinical pregnancy rates in women undergoing assisted reproductive treatments, further emphasizing the importance of maintaining optimal VD levels for reproductive success [49].

Although previous studies have attempted to demonstrate a potential association between VD deficiency and fertility, the results remain inconclusive and controversial. Therefore, further research is necessary to clarify its potential benefits in human reproduction. A study conducted by McGovern et al. (2020) states that VD levels do not have a significant relationship with ongoing pregnancy rates, live births, or miscarriage rates in the context of in vitro fertilization (IVF) [54].

Yang et al. (2025) conducted a systematic review and meta-analysis which involved the analysis of 2583 articles including 66 trials with a total of 17,276 participants to assess the effects of VD supplementation during pregnancy [55]. No significant impact was observed in reducing the risk of preterm birth, low birth weight, neonatal mortality, gestational hypertension, cesarean delivery, or preeclampsia. However, a 35% reduction in the risk of gestational diabetes was reported, along with an increase in maternal and umbilical cord 25(OH)D levels at an average of 29.2 nmol/L. Additionally, a slight increase in birth weight, length, and head circumference was noted [55].

While a direct causal link between VD insufficiency and pregnancy outcomes has not been definitively established, substantial evidence suggests a significant correlation. VD plays a crucial role in ovarian reserve, steroidogenesis, and follicular development. It exhibits anti-inflammatory and immunomodulatory properties by regulating cytokine expression, which is essential for maintaining a favorable reproductive environment [40,41,42,45,46,49].

## 6. Vitamin D Receptor in Reproductive-Associated Tissues

VD’s endogenous production is absorbed from the gut or synthesized in the skin and then converted into 25-hydroxyvitamin D [25(OH)D], mainly by CYP2R1, through hydroxylation in the liver, and into its active form, 1,25-dihydroxyvitamin D (1,25(OH)2D-calcitriol), by CYP27B through 1a-hydroxylation in the renal proximal tubule [56].

VD plays a significant role in fertility, with its importance emphasized by the widespread distribution of VDR in both central and peripheral reproductive tissues in males and females [26]. VDRs have been detected in various reproductive organs and cells, including the hypothalamus, pituitary gland, ovaries, granulosa cells, endometrium, placenta, decidua, testicles, and spermatogenesis cells [57,58]. The human placenta expresses CYP27B1, which encodes the enzyme 1-alpha hydroxylase responsible for converting VD into its active form, calcitriol. This suggests that the placenta can synthesize active VD, which is critical for regulating pregnancy outcomes, immune responses, and placental development [59]. In the pituitary gland, it plays a role in regulating the secretion of hormones involved in ovulation and menstrual cycle control. The presence of VDR in the endometrium suggests that VD may regulate uterine lining preparation for implantation. The endometrium expresses the CYP27B1 gene, which encodes 1-α-hydroxylase, the enzyme responsible for locally converting 25(OH)D3 into its active form, 1,25(OH)2D3 [60,61]. The first study to demonstrate the presence of VDR in human endometrial tissue was conducted by Vienonen et al. (2004), who investigated the expression pattern of nuclear receptors and co-factors in the endometrium using real-time PCR [61]. Notable differences in expression levels were reported among individuals based on age, but no difference in receptor expression was found between the proliferative and secretory phases of the menstrual cycle [62]. Subsequently, a study by Bergadà et al. in 2014 demonstrated that there are indeed differences in VDR expression according to the menstrual cycle, reporting a decrease in total VDR expression in the proliferative endometrium, contrasting with an increase in VDR protein expression during the secretory phase [63]. A systematic review by Cermisoni et al. (2018) confirmed that VDR is expressed in the ovaries, endometrium, and myometrium, highlighting VD’s involvement in fertility and reproductive health [64]. These findings suggest that VD may play a role in endometrial remodeling and preparation for embryo implantation, particularly during the secretory phase.

In male reproductive organs, including the testis, epididymis, and prostate, VDR plays an important role specifically in testicular somatic and germ cells [58]. Testicular somatic and germ cells appear capable of synthesizing and breaking down VD independently of systemic metabolism. Furthermore, VDR expression in the testes indicates that VD may exert autocrine and paracrine effects, potentially contributing to testicular function regulation and influencing male fertility independently of systemic metabolism [63].

These findings collectively underscore the essential role of VD and its receptor in regulating multiple aspects of reproductive function, ranging from steroidogenesis in the ovaries to endometrial receptivity and immune regulation during pregnancy.

## 7. Vitamin D and Fertility

Low serum levels of VD may adversely affect female fertility by influencing ovarian function, implantation, and embryo development. In a study conducted by Pal et al. (2016), it was found that women with VD deficiency exhibited a significantly lower ovarian reserve and an increased risk of anovulation, both of which are critical factors influencing fertility outcomes [65].

Infertility, as defined by the International Glossary on Infertility and Fertility Care, refers to the inability to achieve a clinical pregnancy after 12 months of regular, unprotected intercourse or due to an impairment in reproductive capacity [66]. While the International Society of Endocrinology provides general guidelines for serum VD concentrations, considering levels over 30 ng/mL as optimal, those between 21 and 29 ng/mL as insufficient, and those below 20 ng/mL as deficient, there are currently no precise criteria to define VD deficiency in the context of infertility [67,68]. A prospective cohort study by Jukic et al. (2019) demonstrated that fertility outcomes improve with higher VD levels, with each 10 ng/mL increase in 25-OH-VD correlating to a 10% higher fertilization rate. Women with 25-OH-VD levels below 20 ng/mL experienced a 45% reduction in fertilization rates compared to those with levels between 30 and 40 ng/mL, emphasizing the importance of maintaining optimal VD levels for improved fertility outcomes [69].

Additionally, VD’s role in regulating immune function and reducing inflammation has been implicated in its effect on the uterine environment, particularly in terms of implantation and pregnancy maintenance [70]. VD influences the expression of several genes that are important for embryo development and implantation success, emphasizing its potential as a modulator of reproductive outcomes [71].

Regarding the effects of VD supplementation on the first weeks of pregnancy, the available RCT data have been extensively reviewed in the medical literature. However, findings remain inconclusive due to variability in study design, sample size, and outcome measures [72,73].

A systematic review by Roth et al. (2017) analyzed existing RCTs on prenatal VD supplementation and found that most trials conducted up to that point were small in scale and of low quality, providing insufficient evidence to establish definitive clinical or policy recommendations, highlighting gaps in the effects of VD during early pregnancy [73]. Moreover, since then, new studies have been conducted, like the systematic review and meta-analysis by Yang et al. (2025), which examined 66 RCTs involving 17,276 participants and found that VD supplementation during pregnancy was associated with increased infant birth weight and a potential protective effect against gestational diabetes [55,74]. However, the authors noted high heterogeneity among studies and a considerable risk of bias, limiting the ability to draw strong clinical conclusions [54]. Similarly, a meta-analysis by Liu et al. (2022), which included 42 RCTs, reported that VD supplementation was positively associated with improvements in offspring VD status and some growth parameters [75]. However, the study found no significant effects on birth weight or preterm birth rates, further emphasizing the uncertainty regarding its impact on early pregnancy outcomes [75].

While a substantial number of RCTs have examined VD supplementation during pregnancy, its specific effects during the early weeks remain uncertain due to study limitations. However, existing evidence suggests that VD plays a role in pregnancy outcomes, though the optimal dosage and precise mechanisms influencing implantation, placental development, and pregnancy viability are yet to be fully understood. Future well-designed RCTs should prioritize the first trimester to accurately quantify VD’s role, which will be key to unlocking these answers and optimizing maternal and fetal health outcomes.

In females, VD levels are correlated with AMH levels, a marker of ovarian reserve and function [76]. AMH levels, which fluctuate seasonally, are notably lower in winter compared to summer, with a reduction of up to 18% during the colder months [77]. In males, VD also has a significant impact on testosterone levels, which is age-dependent. In adolescents, no significant relationship between VD and testosterone is observed. However, in older men, higher VD levels are positively correlated with increased testosterone production [78]. VD has also been shown to influence spermatogenesis and sperm motility in the male reproductive system through its interaction with the CYP24A enzyme and the cAMP/PKA signaling pathways [79]. Studies have shown that maternal VD levels affect sperm selection during the fertilization process, facilitating key processes such as capacitation, hyperactivation, and the acrosomal reaction, which are necessary for successful fertilization [47,77]. However, the exact mechanisms through which it exerts these effects remain inadequately understood, highlighting gaps in current research. Regarding the potential of VD supplementation to prevent miscarriage in infertile women, current evidence presents a complex picture. A systematic review and meta-analysis found that women with VD deficiency had an increased risk of miscarriage compared to those with sufficient levels (odds ratio, 1.94; 95% confidence interval, 1.25–3.02) [78]. However, the same analysis noted that whether preconception VD treatment reduces the risk of miscarriage remains unknown. Additionally, a prospective cohort study reported no association between preconception VD levels and miscarriage risk in women conceiving naturally [80]. These findings suggest that while VD deficiency is associated with an increased risk of miscarriage, supplementation’s effectiveness in preventing miscarriage, particularly in infertile women, is not yet conclusive. Further large-scale, high-quality randomized controlled trials are necessary to establish definitive recommendations.

A study by Bacanakgil et al. (2022) revealed that higher serum concentrations of 25-hydroxyvitamin D (25-OH-VD) in women undergoing IVF were associated with better embryo quality and higher clinical pregnancy rates [42]. VD also influences telomere length and telomerase activity, which has been linked to a reduction in chromosomal abnormalities, thereby optimizing IVF outcomes [42].

The “supplementation of VD and reproductive outcome” (SUNDRO) study, which aimed to assess whether VD supplementation could improve IVF outcomes, found no significant difference between the VD supplementation group and the placebo group. Both groups achieved clinical pregnancies, with a slightly higher success rate in the placebo group (40% vs. 37%) [57,81]. However, the confidence intervals for the VD supplementation group were broader, suggesting that VD supplementation did not significantly improve clinical pregnancy rates in this cohort [82].

VD supplementation has been shown to enhance endometrial receptivity by modulating inflammatory markers and improving immune responses [70]. Although these findings are promising, the optimal dosage and duration of VD supplementation in infertile women remain areas of active research, as the therapeutic response may vary depending on individual characteristics such as baseline VD levels and comorbidities [83]. Table 1 emphasizes the most important studies regarding infertility and VD. Also, VD supplementation in patients undergoing intracytoplasmic sperm injection (ICSI) procedures significantly increased the number of top-quality embryos, irrespective of whether patients began with normal or low baseline VD levels. Additionally, in patients with low VD, supplementation improved endometrial thickness, VD levels in follicular fluid, and progesterone levels [76].

## 8. Vitamin D and PCOS

In women with PCOS, VD supplementation has been linked to improved insulin sensitivity and hormonal regulation, further supporting its use as a therapeutic strategy [91]. PCOS is a heterogeneous disease of unclear etiology and one of the most frequent endocrine disorders in females of reproductive age. Its prevalence ranges from 6 to 13%, depending on the diagnostic criteria [91,92]. The American College of Obstetrics and Gynecology (ACOG) defines PCOS as a disorder characterized by hyperandrogenism, ovulatory dysfunction, and polycystic ovaries [93]. The Rotterdam criteria, one of the most widely used diagnostic tools, evaluates oligo- and/or anovulation, clinical or biochemical signs of hyperandrogenism (such as acne and hirsutism), and polycystic ovaries on transvaginal ultrasound, characterized by 20 or more cysts or a volume of 10 mL in any ovary. PCOS may be diagnosed if two of the Rotterdam criteria are present [94].

Functional ovarian hyperandrogenism has been identified as the essential feature of PCOS, with genetic and environmental factors also playing a role. Patients with PCOS often present with abnormal ovarian androgenic function, resulting in elevated levels of insulin resistance, obesity, or luteinizing hormone (LH). Ovarian hyperandrogenism, combined with insulin-resistant hyperinsulinism, is considered the most significant factor in PCOS pathophysiology, followed by increased LH levels and obesity [95].

VD supplementation has shown promise in experimental models and clinical studies (Table 2). VD has been found to decrease androgen levels and endometrial thickness in experimental PCOS rat models [96]. A randomized controlled trial demonstrated that ovulation in women with PCOS was directly related to VD levels, with higher VD levels increasing the probability of ovulation from 68% in cases with levels below 20 ng/mL to 78% in cases with levels above 30 ng/mL [65].

Low VD levels have been correlated with follicular arrest in women with PCOS. A study involving 60 women with PCOS found that a combination of VD, calcium, and metformin increased the number of dominant follicles [97,98]. Regarding insulin resistance in PCOS, low VD levels have been linked to metabolic syndrome, exacerbating its symptoms [64]. In a randomized, double-blind, placebo-controlled trial, Maktabi et al. (2017) found that VD supplementation of 50,000 IU decreased fasting plasma glucose (*p* = 0.02), reduced insulin levels (*p* = 0.004), and increased insulin sensitivity (*p* = 0.007) [99]. However, a more recent study found that VD supplementation reduced plasma glucose during an oral glucose tolerance test but had no other significant metabolic effects [100].

Hormones and endocrine markers have also been studied in relation to PCOS and VD. Davis et al. (2018) found that PCOS cases with androgen excess were more prevalent in VD-deficient patients (*p* = 0.06) [101]. Another randomized controlled trial found a significant treatment effect on the LH/FSH ratio (*p* = 0.022) but no significant effects on FSH and AMH levels in women with PCOS [102]. In a systematic review and meta-analysis, serum AMH was found to significantly decrease following VD supplementation in PCOS, although it is a complex relationship. A prospective cross-sectional study found no relationship between AMH levels and VD deficiency [103,104]. This complicated relationship is made even more complicated by variables affecting AMH levels. Seasonal variables that decrease sun exposure, VD intake, and menstrual cycle timing have been found to have an impact [104]. Further factors and the association between AMH and VD must be studied more extensively to be able to understand it.

**Table 2:** Overview of clinical trials on VD and PCOS.

Study	Country	Total Participants (N)	Cases (*n*)	Controls (*n*)	Age	BMI	Baseline 25(OH)D	Type of Study	Conclusions
Trummer et al. (2019) [100]	Austria	N = 123	*n* = 81	*n* = 42	25.9 ± 4.7 years	27.5 ± 7.3 kg/m^2^	48.8 ± 16.9 ng/mL	Randomized controlled trial	VD supplementation resulted in a reduction in plasma glucose after 60 min during the OGTT. Aside from this, it had no significant effect on metabolic and endocrine parameters in PCOS.
Ostadmohammadi et al. (2019) [105]	Iran	N = 60	*n* = 30	*n* = 30	24.9 ± 4.9 years	24.7 ± 4.6 kg/m^2^	-	Randomize, double-blind, placebo-controlled clinical trial	Supplementation of VD and probiotics, when compared to placebo, significantly improved depression, anxiety, stress, general health, and overall well-being in women with PCOS. It also contributed to a decrease in total testosterone, hirsutism, inflammation, and oxidative stress. Furthermore, there was a notable increase in total antioxidant capacity and glutathione levels.
Lerchbaum et al. (2021) [102]	Austria	N = 330	*n* = 180	*n* = 150	30.5 ± 6.9 years	26.5 ± 6.7 kg/m^2^	50.4 ± 19.0 ng/mL	Double-blind randomized controlled trial	VD treatment in women with PCOS significantly affected FSH and the LH/FSH ratio after 24 weeks, but did not impact AMH levels. No significant effects were observed in non-PCOS women.
Dastorani et al. (2018) [106]	Iran	N = 40	*n* = 20	*n* = 20	30.0 ± 3.93 years	28.05 ± 3.31 kg/m^2^	10.75 ± 2.45 ng/mL	Randomize, double-blind, placebo-controlled trial	VD supplementation significantly reduced serum AMH, insulin levels, and insulin resistance (HOMA-IR) while increasing insulin sensitivity (QUICKI) compared to the placebo. Additionally, it led to a significant decrease in total cholesterol and LDL cholesterol levels.
Javed et al. (2019) [107]	United Kingdom	N = 37	*n* = 18	*n* = 19	28.6 ± 6.4 years	34.58 ± 9.01 kg/m^2^	28.32 ± 11.25 ng/mL	Randomized, double-blind, placebo-controlled study	This study shows that VD supplementation has beneficial effects on liver injury and fibrosis markers (ALT levels), along with modest improvements in insulin resistance (HOMA-IR) in overweight and obese VD-deficient women with PCOS. However, no significant changes were seen in other cardiovascular risk factors or hormones.
Azhar et al. (2024) [108]	Pakistan	N = 180	*n* = 120	*n* = 60	33.2 ± 6.7 years	28.1 ± 5.7 kg/m^2^	-	Comparative descriptive study	Women with PCOS had lower VD levels. Both women with PCOS and VD deficiency exhibited lower HDL levels and higher total cholesterol, LDL, VLDL, and triglyceride levels compared to women without PCOS or those with sufficient or insufficient VD levels.
Irani et al. (2015) [109]	United States	N= 53	*n* = 35	*n* = 18	30.2 ± 1.3 years	29.3 ± 1.6 kg/m^2^	-	Randomized placebo-controlled trial	VD supplementation in women with PCOS increased VD levels and led to shorter menstrual cycles, reduced hirsutism (Ferriman–Gallwey score), lower triglycerides, and a decreased TGF-β1-to-sENG ratio, highlighting VD’s potential role in improving lipid metabolism and inflammation in PCOS.
Wen et al. (2024) [110]	China	N = 57	*n* = 28	*n* = 29	26.0 ± 8.9 years	25.0 ± 7.6 kg/m^2^	12.7 ± 4.7 ng/mL	Randomized controlled trial	VD supplementation increased serum 25(OH)D levels. After 12 weeks, women in the VD group had lower BMI, WHR, insulin, HOMA-IR, triglycerides, total cholesterol, and LDL-C compared to the control group in women with obesity or insulin resistance.
Krul-Poel et al. (2018) [111]	Netherlands	N = 1088	*n* = 639	*n* = 449	33.2 ± 5.1 years	24.7 ± 5.4 kg/m^2^	55.4 ± 35.5 ng/mL	Cross-sectional comparison study	VD deficiency in PCOS may contribute to metabolic issues, especially insulin resistance and lipid abnormalities. Women with PCOS had lower VD levels, which were associated with higher insulin resistance (HOMA-IR) and poorer lipid profiles, including reduced HDL-cholesterol and apolipoprotein A1.

## 9. Vitamin D and Endometriosis

Endometriosis is a chronic inflammatory disease dependent on estrogen, characterized by the presence of endometrium-like tissue in extrauterine locations (columnar epithelium and stroma), affecting 10% of women in reproductive age [112]. This ectopic tissue can be located in pelvic organs such as the bladder, ovaries, fallopian tubes, intestines, and abdominal wall. However, its presence has also been reported in extra-pelvic organs, such as the central nervous system (CNS), pleura, and pericardium [113,114]. Endometriosis is accompanied by clinical manifestations such as chronic pelvic pain, dysmenorrhea, dyspareunia, bladder or intestinal dysfunction, and infertility [115].

The pathophysiology of endometriosis has not been fully elucidated; however, multiple theories have been proposed to explain the disease. One of the most recognized is the theory of retrograde menstruation, first proposed by Sampson, which suggests that menstrual blood does not flow outward through the vaginal canal, but instead travels through the fallopian tubes and disperses into various regions of the pelvic cavity. This process allows fragments of endometrial tissue to adhere to peritoneal organs, leading to lesion formation; the theory has lost validity over time as it fails to explain the presence of endometriosis in extra-pelvic organs or in male patients [114].

On the other hand, the theory of Mülleriosis suggests that the disease is predetermined during embryonic development due to abnormalities in cellular differentiation or migration of the Müllerian duct system. During embryogenesis, the coelomic epithelium, derived from the external mesoderm and a precursor of female reproductive organs, may undergo abnormal migration outside the uterus, promoting the displacement of Müllerian-derived tissues and contributing to the formation of ectopic endometrial lesions [113,114].

Additionally, the Homeobox (HOX) gene theory proposes that the abnormal migration of endometrial stem cells, followed by their activation, plays a fundamental role in the pathogenesis of the disease. This hypothesis is particularly relevant as it provides a more comprehensive explanation for the diverse ectopic locations of endometriotic lesions and their manifestation in male patients [113,114,116].

The prevalence of endometriosis varies significantly depending on the population and diagnostic methods used. Globally, it affects 5–15% of women in reproductive age, with a peak incidence between 25 and 45 years. However, it is also observed in adolescents and, less commonly, in postmenopausal women (2–5%) [114]. Prevalence estimates range from 2 to 11% in asymptomatic women, 5 to 50% in infertile women, and 5 to 21% in those hospitalized for pelvic pain [114,117]. In the U.S., incidence rates are highest in women aged 25–34 years, reaching up to 380.6 cases per 100,000 person-years [117,118]. In Mexico, insufficient emphasis has been placed on this pathology, making it challenging to obtain reliable epidemiological data. However, it is estimated that 34.5% of infertility cases are associated with endometriosis. Moreover, a higher prevalence has been observed in reproductive-age patients with Müllerian malformations [119].

The conventional imaging diagnosis of endometriosis is primarily performed using transvaginal ultrasound (TVUS), high-quality transvaginal ultrasound (HQ-TVUS), and abdominopelvic ultrasound. In cases of deep infiltrating endometriosis, magnetic resonance imaging (MRI) is required for a more accurate assessment [120]. However, a more precise diagnostic method known as endometriosis mapping has been recently introduced. This approach involves comparing imaging results, such as abdominal MRI, with the ENZIAN classification, and correlating them with surgical findings or exploratory laparoscopy outcomes in the patient [121].

The treatment of endometriosis depends on its location, extent, and the patient’s age. In most cases, the primary approach is therapeutic, aiming to reduce or eliminate lesions through surgical intervention, combined with pharmacological treatments to prevent disease recurrence [118]. Patient autonomy is crucial in determining the most appropriate treatment plan. For individuals seeking to preserve fertility, it is essential to avoid damage to organs such as the uterus and ovaries. In these cases, less invasive treatments should be prioritized, including medications to alleviate symptoms and hormonal therapies to suppress endometrial tissue growth and inflammation [114,122].

Given its association with anti-inflammatory, immunomodulatory, and anti-proliferative processes, VD has been emphasized as a potential supplement for the treatment and symptomatic improvement of endometriosis, as it may enhance cellular apoptosis and reduce inflammation [7,68]. VDR is expressed in the healthy human endometrium and myometrium, as well as in patients with endometriosis, specifically in peritoneal endometriotic lesions. Moreover, immune cells have been shown to be present within endometrial lesions [123]. Additionally, a study by Delbandi et al. (2021) analyzed serum levels of 25(OH)D in Iranian women, including 56 healthy participants and 54 patients with endometriosis. The study found that patients with serum 25(OH)D levels below 20 ng/mL had a 2.7-fold higher risk of developing endometriosis compared to women with higher 25(OH)D levels [124,125].

Nodler et al. (2020) conducted a randomized trial involving 69 adolescent women aged 12–25 years, who were divided into three groups: 20 received fish oil supplements, 22 received a placebo, and 27 received VD supplementation. Among the three groups, the most significant improvement over a six-month period was observed in patients supplemented with VD, as reflected in the Visual Analog Scale (VAS) for pain. Initially, participants had an average VAS score of 7.0, which decreased to an average of 5.5 in the final month of the study [7,125].

Zheng et al. (2023) performed a systematic review and meta-analysis on the use of antioxidants such as VD, vitamin E (VE), and vitamin C (VC) to evaluate their effectiveness in reducing pelvic pain in patients with endometriosis [126]. Their findings indicated that VD significantly reduces dysmenorrhea over a 12-month period when administered at a dose of 50,000 IU weekly or biweekly. However, VD did not show a significant improvement in dyspareunia compared to the placebo group [126]. Table 3 highlights the findings regarding VD and endometriosis.

## 10. Discussion

VD deficiency among women of reproductive age constitutes a significant public health concern, driven by factors such as inadequate dietary intake, limited sun exposure, and lifestyle changes. National supplementation programs, such as Denmark’s margarine fortification initiative, demonstrated reproductive benefits, including improved live birth rates in women diagnosed with infertility. These findings underscore the value of population-level interventions to address VD deficiency as a cost-effective strategy for enhancing reproductive outcomes [67,68].

A randomized controlled trial by Pal et al. (2016) demonstrated that higher VD levels were associated with a significant increase in ovulation rates among women with PCOS [65]. This study, while providing valuable evidence, has limitations, including a relatively small sample size and a focus on a narrow patient group, which may limit the generalizability of its findings. Additionally, it did not establish an optimal dose of VD supplementation for maximal reproductive benefit. In contrast, a larger study by Maktabi et al. (2017) also found improvements in insulin sensitivity and metabolic markers in PCOS patients following VD supplementation, reinforcing the potential of VD in managing the metabolic aspects of PCOS [99]. However, the absence of consistent effects on other reproductive outcomes, such as follicular development and ovulation, suggests that VD’s impact may be more significant for managing metabolic dysregulation than for directly influencing fertility. This variability between studies underscores the need for more tailored research to identify which aspects of PCOS are most responsive to VD supplementation.

On the other hand, studies involving VD supplementation in women with endometriosis have yielded more inconsistent results. While some studies have reported a reduction in endometriotic lesion size and improved pain management with VD supplementation, others have failed to demonstrate significant clinical benefits [123,125]. These discrepancies may be attributed to differences in study methodologies, such as varying VD dosages, treatment durations, and patient populations. Moreover, the underlying pathophysiology of endometriosis is complex, and the mechanisms through which VD could influence disease progression are not fully understood. While VD’s potential anti-inflammatory properties and its role in immune modulation provide a plausible mechanism, further investigations are needed to establish definitive clinical guidelines for VD use in managing endometriosis.

Current research on VD’s role in reproductive health, while promising, is hindered by several limitations. One of the most significant challenges is the variability in VD supplementation protocols across studies. For instance, different studies have employed varying doses of VD (ranging from 1000 IU to 50,000 IU daily), without a consensus on the optimal dose for improving fertility outcomes [78]. Additionally, the duration of supplementation varies widely, with some studies using short-term regimens (e.g., 8–12 weeks) and others extending over several months. This inconsistency makes it difficult to draw definitive conclusions about the efficacy of VD supplementation in improving reproductive health outcomes [85].

A systematic review by Kalaitzopoulos et al. (2022) reported that in women with primary dysmenorrhea, a single dose of 300,000 IU of VD taken five days before menstruation reduced pain intensity [123]. Regarding VD supplementation, a regimen of 50,000 IU weekly for six weeks before embryo transfer did not significantly improve pregnancy rates [123]. For endometriosis, a dose of 50,000 IU of VD every 2 weeks for 12 weeks was found to reduce pelvic pain and lower C-reactive protein levels [132]. Additionally, a study by Nodler et al. (2020) supplemented adolescents diagnosed with endometriosis with 2000 IU of VD daily for six months and reported improvements in pelvic pain [7].

Regarding PCOS, guidelines recommend daily doses of 2000 to 4000 IU of VD, as 67–85% of these patients are VD-deficient, until normal VD levels are reached [26]. A meta-analysis by Yang et al. (2023) reported that the pregnancy and ovulation rates were significantly higher in patients who received weekly doses of 5000 IU of VD, and that VD supplementation also reduced testosterone levels in women with PCOS [133]. Additionally, Mohan et al. (2023) reported that VD deficiency negatively affects reproductive capacity, suggesting that VD supplementation could improve fertility rates [11].

Moreover, many studies have small sample sizes and lack long-term follow-up, limiting the robustness of their findings. Larger, multicenter trials are needed to confirm these findings and to establish the long-term effects of VD supplementation on fertility, especially in women with reproductive disorders such as PCOS and endometriosis.

Another challenge is the lack of standardized VD deficiency thresholds and treatment protocols. Studies often define VD deficiency differently, and the lack of universally accepted guidelines for VD levels in fertility and reproductive disorders complicates the interpretation and comparison of results. For example, while some studies use a threshold of 20 ng/mL for VD deficiency, others set it at 30 ng/mL, which may influence the observed outcomes [67,68]. These discrepancies highlight the need for future research to standardize VD deficiency criteria and supplementation regimens to ensure consistency across clinical studies. Different age groups and populations have varying VD requirements. These include infants and children under the age of four, pregnant and breastfeeding women, particularly adolescents and young women, people over the age of 65, people who have limited access to sunlight due to cultural practices, people who live at home or spend extended periods indoors, and people with darker skin tones, among other factors.

Despite these limitations, the evidence suggests that VD supplementation could hold significant promise as a therapeutic strategy for improving fertility outcomes in women with PCOS, endometriosis, and other reproductive health disorders.

## 11. Conclusions

VD deficiency is a major public health challenge for women of reproductive age due to its implications for fertility. The scientific literature suggests that VD supplementation can enhance fertility outcomes, particularly in conditions such as endometriosis and PCOS. The primary benefit of VD supplementation appears to be its role in metabolic regulation, which in turn influences reproductive function. Additionally, some studies have demonstrated positive effects on metabolic markers and ovulation in both endometriosis and PCOS. However, the absence of standardized criteria for diagnosing VD deficiency and the lack of consensus on optimal supplementation regimens considering dosage, treatment duration, and individual patient needs remain significant limitations. Despite these challenges, VD supplementation emerges as a promising therapeutic strategy for improving reproductive health, particularly in individuals with documented deficiencies.

Despite these challenges, VD supplementation emerges as a promising therapeutic strategy for improving reproductive health, particularly in individuals with documented deficiencies. However, the heterogeneity in study designs, VD, and patient populations limits the ability to establish definitive clinical guidelines. Future research should prioritize large-scale, well-controlled randomized trials to determine the optimal VD levels and supplementation strategies for improving reproductive outcomes. Additionally, further studies exploring the molecular mechanisms of VD in ovarian function, implantation, and inflammation could provide deeper insights into its therapeutic potential. From a clinical perspective, assessing VD status in patients with reproductive disorders and considering supplementation as part of a personalized treatment approach may enhance fertility and overall reproductive health. More research is needed to define the optimal intake, duration, and long-term effects of VD therapy in these populations.

It is important to acknowledge that, as a narrative review, this work does not follow the strict methodological framework of a systematic review or meta-analysis. While this approach allows for a broader synthesis of available evidence, it also introduces potential limitations, such as selection bias and the absence of quantitative analysis. Future systematic reviews and meta-analyses are needed to provide stronger evidence through standardized selection criteria and statistical comparisons. Nonetheless, this review offers valuable insights into the current understanding of VD in reproductive health and highlights the need for further investigation in this field.

## Figures and Tables

**Table 1:** Overview of impactful studies on VD and fertility.

Study	Country	Total Participants (N)	Cases (*n*)	Controls (*n*)	Age	BMI	Baseline 25(OH)D	Type of Study	Conclusions
Aflatoonian et al. (2014) [84]	Iran	N = 114	*n* = 57	*n* = 57	29.0 ± 4.3 years	26.58 ± 1.72 kg/m^2^	15.02 ± 6.14 ng/mL	Randomized clinical trial	VD insufficiency treatment did not significantly improve pregnancy rates in frozen-thawed embryo transfer cycles.
Bezerra Espinola et al. (2020) [85]	Italy	N = 100	*n* = 50	*n* = 50	35.30 ± 5.41 years	21.95 ± 2.20 kg/m^2^	23.40 ± 6.65 ng/mL	Prospective randomized controlled pilot study	Vitamin D3 supplementation (2000 IU/day) improved implantation rates in IVF patients (37.1% vs. 19.2%, *p* = 0.0151), particularly in those with baseline VD ≥ 20 ng/mL. No significant difference in ongoing pregnancy or miscarriage rates.
Doryanizadeh et al. (2020) [86]	Iran	N = 95	*n* = 51	*n* = 44	32.08 ± 4.90 years	25.11 ± 3.29 kg/m^2^	27.55 ± 1.80 ng/mL	Double-blind randomized clinical trial	Calcitriol significantly increased chemical pregnancy rates (31.4% vs. 18.2%, *p* < 0.05), but had no significant effect on clinical pregnancy (25.5% vs. 13.6%) or ongoing pregnancy at 20 weeks (9.8% vs. 11.6%).
Somigliana et al. (2021) [87]	Italy	N = 630	*n* = 308	*n* = 322	35.0 ± 3.9 years	21.5 ± 2.3 kg/m^2^	20.0 ± 5.1 ng/mL	Double-blind randomized controlled trial	A single high dose (600,000 IU) of vitamin D3 before IVF did not improve clinical pregnancy rates (37% vs. 40%, *p* = 0.37) or IVF outcomes. No specific subgroup benefited from supplementation. Further studies are needed.
Anifandis et al. (2010) [88]	Greece	N = 101	*n* = 21	*n* = 80	36.16 ± 5.29 years	24.10 ± 2.99 kg/m^2^	23.37 ± 8.45 ng/mL	Prospective observational study	Higher follicular fluid VD levels (>30 ng/mL) were associated with lower embryo quality and reduced pregnancy rates (14.3% vs. 32.3–32.7%, *p* = 0.047). Increased VD levels correlated with lower follicular fluid glucose levels, possibly affecting oocyte quality and IVF outcomes.
Franasiak et al. (2015) [89]	United States	N = 517	*n* = 208	*n* = 309	35.25 ± 4.27 years	25.04 ± 5.42 kg/m^2^	24.20 ± 4.65 ng/mL	Retrospective cohort study	Vitamin D levels were not associated with IVF success after euploid blastocyst transfer. No significant differences in implantation or pregnancy rates across VD categories. Measuring VD does not predict implantation likelihood in this population.
Fabris et al. (2014) [90]	Spain	N = 267	*n* = 41	*n* = 226	40.78 ± 0.76 years	22.89 ± 0.76 kg/m^2^	>30 ng/mL	Retrospective cohort study	VD levels did not correlate with pregnancy rates in egg donation recipients. No significant differences in implantation (61% vs. 63.4% vs. 65.2%), pregnancy (70% vs. 69.9% vs. 73.9%), or ongoing pregnancy rates (55.9% vs. 52.7% vs. 60.7%). No evidence to recommend VD screening in egg donation patients.
Abedi et al. (2019) [72]	Iran	N = 85	*n* = 42	*n* = 43	31.34 ± 4.30 years	23.85 ± 2.00 kg/m^2^	13.54 ± 6.50 ng/mL	Randomized double-blind placebo-controlled trial	VD supplementation (50,000 IU weekly for 6 weeks before ICSI) significantly improved endometrial quality (81% vs. 55.8%, *p* = 0.015), chemical pregnancy rate (47.6% vs. 25.5%, *p* = 0.013), and clinical pregnancy rate (38.1% vs. 20.9%, *p* = 0.019). No significant effect on oocyte retrieval, fertilization rate, or embryo quality.

**Table 3:** Overview of impactful studies on VD and endometriosis.

Study	Country	Total Participants (N)	Cases (*n*)	Controls (*n*)	Age	BMI	Baseline 25(OH)D	Type of Study	Conclusions
Chamgordani et al. (2024) [127]	Iran	N = 59	*n*= 29	*n*= 30	36.5 ± 5.5 years	26.4 ± 3.9 kg/m^2^	27.3 ± 3.2 ng/mL	Case Control Study	Women with advanced endometriosis had higher NK cell percentages than controls, but no significant correlation was found between NK cells and vitamin D levels. Serum vitamin D levels were slightly higher in endometriosis cases, but the difference was not significant (*p* = 0.12).
Somigliana E et al. (2007) [82]	Italy	N = 140	*n* = 87	*n* = 53	33.7 ± 6.0 years	21.9 ± 3.4	24.9 ± 14.8 ng/mL	Prospective cohort study	Women with endometriosis had higher serum levels of 25-hydroxyvitamin D3. A positive gradient was observed according to disease severity.
Miyashita M et al. (2016) [128]	Japan	N = 76	*n* = 39	*n* = 37	34.3 ± 1.4 years	-	20.2 ± 1.3 ng/mL	Case–control study with in vitro experiments	Lower serum 25(OH)D3 levels were found in patients with severe endometriosis. VD suppressed inflammatory markers and reduced ESC proliferation, suggesting a potential therapeutic role.
Nodler et al. (2020) [7]	United States	N = 149	*n* = 47	*n* = 22	19.67 ± 3.1 years	26.07 ± 5.46 kg/m^2^	35.3 ± 13.9 ng/mL	Randomize, double-blind, placebo-controlled clinical trial	In young women with endometriosis, neither VD nor fish oil significantly reduced pain compared to placebo. The improvement observed in the placebo group highlights the need for further research to understand this effect.
Zheng et al. (2023) [126]	Chinese	N = 589	*n* = 315	*n* = 274	31.47 ± 4.24 years	-	-	Randomized controlled trial	A randomized controlled trial showed that supplementation with antioxidant vitamins, including VD, significantly reduced dysmenorrhea, dyspareunia, and chronic pelvic pain in women with endometriosis. It also improved the overall quality of life.
Mehdizadehkashi et al. (2021) [129]	Iran	N = 60	*n* = 30	*n* = 30	35.2 ± 7.05 years	24.7 ± 3.5 kg/m^2^	-	Randomize, double-blind, placebo-controlled clinical trial	A 12-week supplementation with VD improved pelvic pain, cholesterol ratio, hs-CRP, and TAC levels in endometriosis patients but did not affect other symptoms. Findings on VD-binding protein were inconsistent. Further research is needed to fully understand its effects on endometriosis.
Harris et al. (2013) [130]	United States	N = 70,556	*n* = 1385	*n* = 69,171	35.92 ± 4.25 years	-	-	Prospective cohort study	This cohort showed that plasma 25(OH)D levels were inversely associated with endometriosis. Women with the highest levels of VD had a 24% lower risk of endometriosis than women with the lowest levels.
Xie et al. (2024) [131]	Germany	N = 3232	*n* = 257	*n* = 2975	38.43 ± 9.57 years	27.32 ± 4.5 kg/m^2^	21.36 ± 10.01 ng/mL	Observational cross-sectional study	Findings were that higher serum 25(OH)D concentrations were associated with a decreased incidence of endometriosis, with data suggesting that adequate sun exposure may lower risk for VD deficiency.
